# Association of depressive and/or pregnancy-related anxiety symptoms in pregnancy with maternal and neonatal biologic aging

**DOI:** 10.1186/s12884-026-08810-1

**Published:** 2026-02-17

**Authors:** Danielle M. Panelli, Katherine Bianco, Sheryl L.Rifas-Shiman, Emily Oken, Marie-France Hivert, Ixel Hernandez-Castro, Gary M. Shaw, Andres Cardenas

**Affiliations:** 1 Division of Maternal-Fetal Medicine and Obstetrics, Department of Obstetrics and Gynecology, Stanford University, Stanford, CA USA; 2 Division of Chronic Disease Research Across the Lifecourse, Department of Population Medicine, Harvard Medical School and Harvard Pilgrim Health Care Institute, Boston, MA USA; 3 Department of Epidemiology and Population Health, Stanford University, Stanford, CA USA; 4 Department of Pediatrics, Stanford University, Stanford, CA USA

**Keywords:** Biologic aging, Telomeres, Mitochondria, Pregnancy, Mental health, Depression, Anxiety

## Abstract

**Background:**

Prenatal depression and anxiety affect nearly 20% of individuals, and emerging evidence links psychological distress to accelerated biological aging. However, the joint effects of depressive and anxiety symptoms on biological aging in mother-infant dyads are understudied. We investigated associations of prenatal maternal depressive and/or pregnancy-related anxiety symptoms with two markers of biological aging [leukocyte telomere length (LTL) and mitochondrial DNA copy number (mtDNAcn)], in maternal blood and neonatal cord blood.

**Methods:**

This was a secondary analysis of a prospective cohort of mother-infant dyads enrolled 1999–2002 during their first prenatal visit to a multispecialty practice in Massachusetts. Participants were eligible if they were carrying a singleton gestation, English-speaking, and under 22 weeks gestational age. The exposure was prenatal depressive symptoms (Edinburgh Postnatal Depression Scale [EPDS] ≥ 13) and/or pregnancy-related anxiety (PrAS moderate-high), grouped into four mutually exclusive categories: 1) low anxiety and low depressive symptoms (referent); 2) high depressive symptoms alone; 3) moderate-high PrAS alone; 4) and both. The primary outcome was maternal and neonatal markers of biological aging and oxidative stress (LTL and mtDNAcn), measured from maternal (1st or 2nd trimester) and neonatal (cord) blood and calculated using quantitative PCR. Multivariable linear generalized estimating equation regression models were used to assess the relationship between prenatal mental health symptoms and maternal/neonatal biological aging/oxidative stress, adjusting for confounders.

**Results:**

Among 751 pregnancies, 479 (64%) had low pregnancy-related anxiety and low depressive symptoms, 33 (4%) had high depressive symptoms alone, 209 (28%) had moderate-high PrAS alone, and 30 (4%) had both. High depressive symptoms alone and moderate-high PrAS were each associated with shorter maternal LTL [ß = -0.07, 95% Confidence Interval (CI) -0.13, -0.02 for depressive; ß = -0.04, 95% CI -0.08, -0.01 for PrAS]. Mothers with depressive symptoms alone also had higher neonatal cord blood mtDNAcn (ß = 0.11, 95% CI 0.01, 0.21).

**Conclusions:**

Prenatal symptoms of depression or pregnancy-related anxiety were independently associated with molecular signatures of accelerated biologic aging in mothers and their neonates. These findings underscore that perinatal mental health symptoms exert measurable biological effects that could influence long-term health trajectories, and reinforce the urgent need for comprehensive mental health care during pregnancy.

**Supplementary Information:**

The online version contains supplementary material available at 10.1186/s12884-026-08810-1.

## Introduction

Perinatal mental health disorders such as depression and anxiety affect approximately 20% of individuals in pregnancy worldwide, and are increasingly recognized as leading contributors to maternal morbidity and mortality [1–5]. Despite growing recognition of perinatal mental health concerns, these conditions remain underdiagnosed, undertreated, and understudied in terms of their clinical and biological consequences [5–9].

Emerging evidence links perinatal psychological distress to molecular biomarkers of accelerated aging, including shorter leukocyte telomere length (LTL) and elevated mitochondrial DNA copy number (mtDNAcn) [10–16]. Shorter LTL and elevated mtDNAcn have been linked with increased health complications across the lifespan in non-pregnant adults [10, 17]. While less studied in pregnancy, these biologic aging and oxidative stress markers have been associated as both predictors and consequences of pregnancy complications, including preeclampsia, preterm birth, and fetal growth restriction [18–24]. However, most prior research on these stress-related biomarkers in the prenatal period has focused on fetal programming effects – how maternal stress during pregnancy alters molecular signatures in the neonate [11]. In contrast, few studies have evaluated effects on maternal biologic aging or considered dyadic mother-infant outcomes.

This knowledge gap is important, as pregnancy itself is a biologic stressor. Mental health conditions may compound this burden, accelerating aging and oxidative stress processes in ways that are currently poorly understood [10, 25, 26]. Moreover, though often co-occurring, depression and anxiety may exert distinct effects on biologic aging pathways during pregnancy; despite this, they are often studied together or without validated measures (with literature particularly sparse for perinatal anxiety) [27]. The issue regarding biological ramifications of active, untreated mental health symptoms takes on added urgency in light of the recent United States Food & Drug Administration Expert Panel about the effects of antidepressant use in pregnancy, which took place on July 21, 2025 [28].

In our study, we investigated whether maternal symptoms of depression and/or pregnancy-related anxiety during pregnancy were associated with accelerated biological aging and increased oxidative stress, measured by short LTL and mtDNAcn abundance, in maternal and neonatal blood samples. We hypothesized that each condition would independently associate with shorter LTL and elevated mtDNAcn, with the strongest effects in individuals who had both high depressive symptoms and moderate-severe pregnancy-related anxiety.

## Materials and methods

### Study design and population

We used data from Project Viva, a pre-birth cohort enrolled between 1999 and 2002 [29]. Pregnant women who presented to care at a multispecialty group practice in Eastern Massachusetts for their initial prenatal visit were eligible for enrollment in Project Viva if they were carrying a singleton gestation, English-speaking, under 22 weeks gestational age, and planning to deliver locally. For the present study, we included pregnant Project Viva participants who completed mental health questionnaires at early and mid-pregnancy visits and had biomarker measurements from maternal blood samples collected during gestation. We excluded participants from all analyses where maternal samples were flagged as contaminated or compromised (e.g. samples where variation between triplicate runs fell outside the threshold) [30]; for the neonatal analyses, we also excluded neonatal cord blood samples that were flagged as poor quality or compromised.

### Exposure

The exposure was prenatal maternal mental health symptoms, assessed via the Edinburgh Postnatal Depression Scale (EPDS) and the Pregnancy-related Anxiety Scale (PrAS) between 5 and 39 weeks of gestation. The EPDS is a 10-item questionnaire scored from 0–30 that is commonly used to screen for perinatal depression, with sensitivities of 59–100% and specificities of 49–100% for clinical diagnosis of depression reported in the literature [31]. Higher scores correlate with greater depressive symptoms; cutoffs between 8 and 13 indicate higher risk of depression. For this study, we defined high depressive symptoms as EPDS ≥ 13 to optimize specificity [32].

The PrAS that was administered at the time of this study was a 7-item survey with 4-point Likert scale responses regarding frequency of the specific symptoms (ranging from not at all to very much) [33, 34]. The PrAS questions assessed level of worry about fetal development, childbirth, loss of baby, and pregnancy complications. We derived three pregnancy-related anxiety categories from the number of “very much” responses out of the 7 questions: “low” if 0/7, “moderate” if 1–2/7, and “high” if ≥ 3/7. For simplicity, pregnancy-related anxiety obtained from the PrAS may occasionally be referred to as “anxiety”, but we acknowledge that this measure is distinct from clinical anxiety. The PrAS was used as it was the only anxiety-related measure collected among the pregnant cohort at the time enrollment procedures were conducted.

Using these PrAS and EPDS definitions of anxiety and depressive symptoms, we generated four mutually exclusive mental health exposure categories: low anxiety (PrAS = 0) and low depressive symptoms (EPDS < 13) used as a referent group, high depressive symptoms alone, moderate-high anxiety alone, and a combination of both moderate-high anxiety plus high depressive symptoms.

### Outcomes

The outcomes were LTL and mtDNAcn, derived from 1 st or 2nd trimester maternal blood (5.7—20.8 weeks gestation) and from neonatal cord blood collected at delivery. Clinicians collected venous cord blood via venipuncture at delivery in the subset of patients who delivered at 1 of 2 study delivery hospitals. We centrifuged all samples within 24 h and stored white blood cells at −80°C prior to assay. The methods for centrifugation, DNA extraction, and telomere length analyses have been previously described for this cohort [30]. Briefly, DNA was extracted from nucleated cells using Puregene Kits (Fisher, Catalog Nos. A407-4, A416-4; Qiagen, Catalog Nos. 158908, 158912, 158924). LTL was measured using quantitative PCR (qPCR) to compare LTL copy number to a single gene copy number (reported as T/S ratio), according to methods described by Cawthon et al. [35] All samples were run in triplicate with standards across plates. Unique to this analysis, the T/S ratio is interpreted as the samples LTL relative to the population average of both maternal and cord blood LTL values in this cohort; therefore, T/S values > 1 are consistent with longer LTL and values < 1 are consistent with shorter LTL than the study mean [30].

Mitochondrial abundance measured as mtDNAcn was quantified from the same maternal and neonatal blood samples as LTL. Similarly, qPCR was used according to the methods of Andreu et al. [36] to calculate the relative amplification of nuclear and mitochondrial segments of DNA. As previously described [30], samples were run in triplicate with standard reference samples (from a tissue-specific pooled sample of all maternal and cord blood samples in the cohort) across plates. Like LTL, the mtDNAcn for each sample is interpreted as the mtDNAcn relative to the population average for both maternal and cord blood in the entire Project Viva study cohort. Therefore values > 1 indicate greater than average mtDNAcn and values < 1 indicate lower than average mtDNAcn.

### Covariates

We collected sociodemographic and clinical characteristics using chart review, questionnaires, and interviews during study visits. Variables of interest included maternal age at enrollment, pre-pregnancy body mass index (BMI), self-reported race and ethnicity, annual household income, highest level of education, parity, active smoking during early pregnancy, gestational age at delivery, infant sex, infant birthweight for age and sex *z*-scores, clinical diagnosis of major depressive disorder prior to mid-pregnancy (in response to question “Have you ever been diagnosed with depression by a health care professional?”), and whether an antidepressant medication was used between the last menstrual period and time of childbirth. We selected confounders for the models using Directed Acyclic Graphs, accounting for potential associations identified in prior research [30, 37–41]. For the maternal models, we selected maternal age, pre-pregnancy BMI, nulliparity, education and income levels, pregnancy smoking, race and ethnicity as social constructs, and analytic sample plate for each biomarker as potential confounders. For the neonatal models, we selected maternal age, pre-pregnancy BMI, nulliparity, education and income levels, and pregnancy smoking, and infant gestational age at delivery, sex, and sex-specific birthweight for gestational age z-score, and analytic sample plate for each biomarker. While neonatal birthweight could be a potential mediator, we chose to include it in the models as it also could be a potential confounder when used as a proxy for estimated gestational weight and given literature highlighting its influence on neonatal stress biomarkers [42, 43].

### Statistical analysis

We compared sociodemographic and clinical characteristics across the four mental health exposure groups using Kruskal–Wallis tests for continuous variables and Chi Square for categorical. For the primary analyses, we assumed pregnancy to be a cross-sectional timepoint with biological plausibility supporting the hypothesis that mental health symptoms would cause biomarker changes rather than the reverse, and that both the mental health symptoms and the biomarkers were likely reflective of more longstanding exposures. We assessed associations of prenatal mental health symptoms with the biological aging biomarkers using linear generalized estimating equation regression models, adjusting for the confounders described above and accounting for clustering of multiple separate births to one woman (*N* = 7 who had two births in the Viva cohort). We ran separate models with each maternal and neonatal biomarker as an outcome, making a total of four models (maternal LTL, maternal mtDNAcn, neonatal LTL, neonatal mtDNAcn). We conducted sensitivity analyses as follows: 1) restricting to women not using antidepressant medications, as these medications may play a complex role in the biologic response to symptoms; 2) restricting to first births, so that all pregnancies were to unique mothers; 3) restricting to pregnancies where both the PrAS and EPDS were completed prior to 28 weeks gestation; and 4) using the EPDS and PrAS scores continuously in the models and assessing joint effects with a statistical interaction term. Confounders for the sensitivity analyses were the same as for the primary models. We used SAS OnDemand (Carey, NC) for analyses. *P*-values < 0.05 defined statistical significance. Participants provided written informed consent at enrollment and in follow-up visits, under the original Project Viva Study (approved by the Harvard Pilgrim Health Care Institute Institutional Review Board). The Stanford University Institutional Review Board deemed our present study IRB exempt due to it being a secondary analysis with no identifiable data.

## Results

**Fig. 1:**
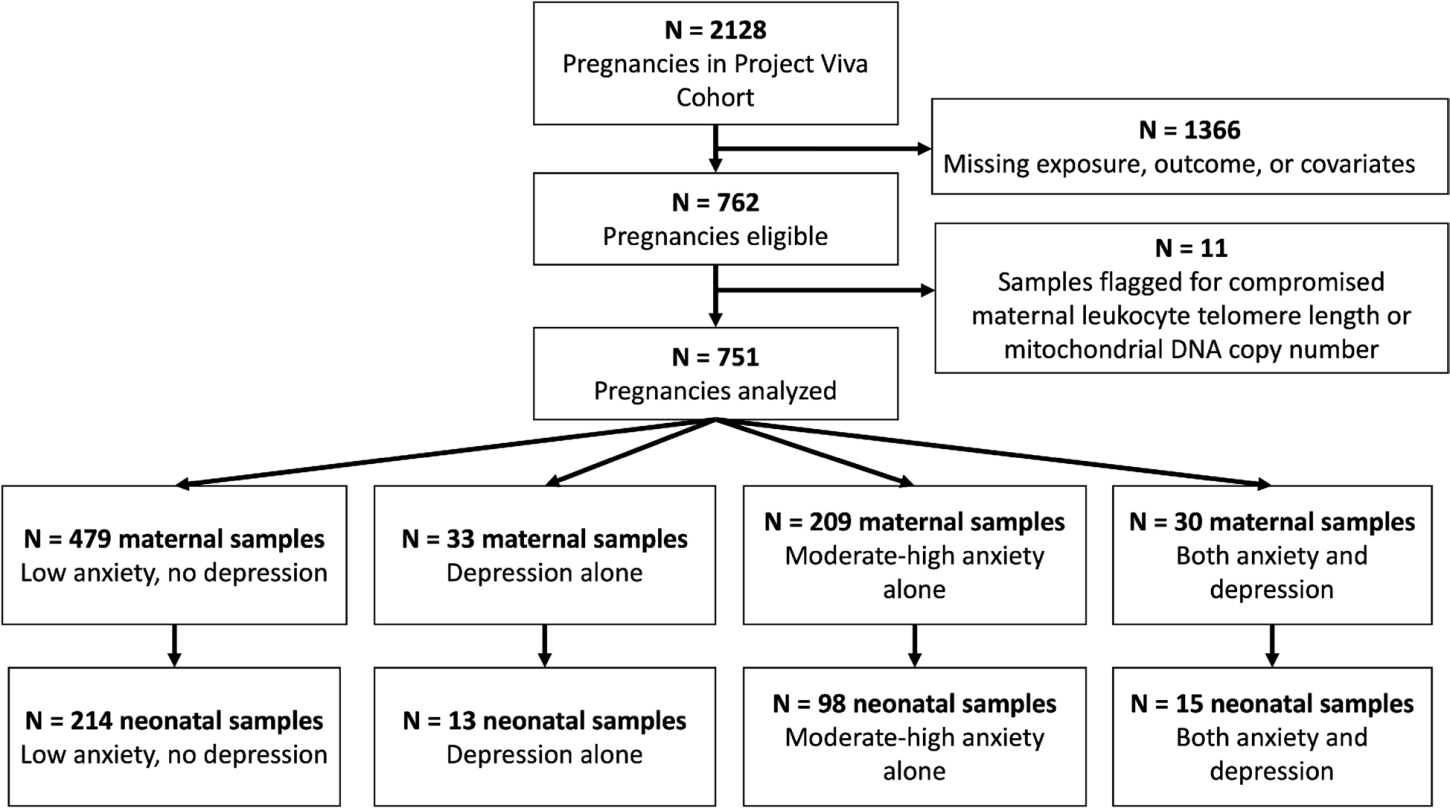
Consort diagram

Of the 2128 pregnancies in the Project Viva cohort with live singleton births, we included 751 pregnancies among 744 unique women in this analysis (Fig. 1). Of the 751 pregnancies, 479 (64%) had low depressive and low pregnancy-related anxiety symptoms (referent group), 33 (4%) had high depressive symptoms alone, 209 (28%) had moderate-high pregnancy-related anxiety alone, and 30 (4%) had both. Sociodemographic characteristics varied across exposure groups, with notable differences for race and ethnicity, income, and parity (Table 1). As expected, more participants with EPDS scores ≥ 13 received an antidepressant medication during pregnancy (15.2% in depressive symptoms alone and 10.0% in combined anxiety/depression group versus 2.4% with moderate-high anxiety alone and 3.8% with neither, *p* < 0.01). EPDS and PrAS scores, prenatal timing of EPDS and PrAS administration, and mental health histories are shown across the 4-level exposure categories in Table 2. All participants had EPDS assessed after their blood draw (in either the 2nd or 3rd trimesters during the study’s Mid-Pregnancy Visit), while all had the PrAS done at the time of the blood draw with other enrollment procedures (at the study’s Early Pregnancy Visit). Demographics were also compared between Project Viva participants who were included versus excluded from this analysis (Supplementary Table 1) and among those for whom neonatal results were available (Supplementary Table 2).

**Table 1:** Demographic and clinical characteristics among study participants enrolled in Project Viva, 1999–2002 (*N* = 751)

	**Total *N* = 751**	**Low PrAS, EPDS < 13**^**a**^ ***N* = 479**	**EPDS ≥ 13 alone** ***N* = 33**	**Moderate-high PrAS alone** ***N* = 209**	**Both moderate-high PrAS and EPDS ≥ 13** ***N* = 30**	***p*-value**^**b**^
Median maternal age (years)	32.5 (29.8, 35.7)	32.8 (30.1, 35.9)	32.3 (28.7, 35.3)	32.2 (29.6, 35.8)	31.1 (29.4, 33.5)	0.18
Median pre-pregnancy body mass index (kg/m^2^)	23.5 (21.3, 26.7)	23.5 (21.3, 26.4)	23.7 (21.3, 27.5)	23.2 (20.8, 27.5)	25.5 (22.4, 27.6)	0.30
Self-reported race and ethnicity						< 0.01
Asian	30 (4.0%)	14 (2.9%)	3 (9.1%)	11 (5.3%)	2 (6.7%)	
Black	67 (8.9%)	30 (6.3%)	4 (12.1%)	24 (11.5%)	9 (30.0%)	
Hispanic	45 (6.0%)	20 (4.2%)	1 (3.0%)	18 (8.6%)	6 (20.0%)	
White	580 (77.2%)	399 (83.3%)	21 (63.6%)	148 (70.8%)	12 (40.0%)	
More than 1 race/ethnicity	29 (3.9%)	16 (3.3%)	4 (12.1%)	8 (3.8%)	1 (3.3%)	
Annual income > $70,000	485 (64.6%)	326 (68.1%)	17 (51.5%)	132 (63.2%)	10 (33.3%)	< 0.01
College graduate	565 (75.2%)	373 (77.9%)	21 (63.6%)	153 (73.2%)	18 (60.0%)	0.04
Nulliparous	390 (51.9%)	228 (47.6%)	14 (42.4%)	135 (64.6%)	13 (43.3%)	< 0.01
Smoked tobacco early in pregnancy	87 (11.6%)	49 (10.2%)	6 (18.2%)	29 (13.9%)	3 (10.0%)	0.34
Median gestational age at maternal blood draw	9.6 (8.7, 10.7)	9.6 (8.7, 10.9)	9.4 (8.4, 11.1)	9.6 (8.7, 10.6)	9.2 (8.6, 10.0)	0.63
Median gestational age at delivery (weeks)	39.9 (38.9, 40.7)	39.9 (39.0, 40.7)	39.7 (38.9, 40.5)	39.9 (38.7, 40.9)	39.8 (39.1, 40.6)	0.97
Median birthweight for age and sex (z-score)	0.2 (−0.4, 0.9)	0.2 (−0.3, 0.9)	0.3 (−0.5, 1.0)	0.1 (−0.5, 0.7)	0.1 (−0.5, 0.7)	0.08
Female infant	374 (49.8%)	238 (49.7%)	16 (48.5%)	106 (50.7%)	14 (46.7%)	0.98

^a^Shown as median (Q1, Q3) or N (column percent). Edinburgh Postnatal Depression Scale (EPDS). Pregnancy-related Anxiety Score (PrAS). See text for details

^b^Kruskal Wallis test for continuous variables, Chi Square for categorical

**Table 2:** Pregnancy-related Anxiety Scores (PrAS), Edinburgh Postnatal Depression Scale (EPDS) scores, and mental health history, by 4-level exposure category (*N* = 751)

	**Low anxiety, EPDS < 13**^**a**^ ***N* = 479**	**EPDS ≥ 13** **alone** ***N* = 33**	**Moderate-high anxiety alone** ***N* = 209**	**Both anxiety and EPDS ≥ 13** ***N* = 30**	***p*-value**^**b**^
PrAS score					
Low	479 (100%)	33 (100%)	0	0	< 0.01
Moderate	0	0	158 (75.6%)	20 (66.7%)	
High	0	0	51 (24.4%)	10 (33.3%)	
Median EPDS score	4 (1,7)	14 (13, 16)	4 (2, 7)	17 (14, 19)	< 0.01
Median gestational age at assessment					
PrAS	9.6 (8.7, 10.7)	9.4 (8.4, 11.1)	9.6 (8.7, 10.6)	9.1 (8.6, 10.0)	0.42
EPDS	27.7 (26.7, 28.6)	27.4 (26.1, 28.3)	27.9 (26.6, 28.7)	27.1 (26.3, 28.3)	0.17
Depression diagnosis prior to mid-pregnancy	63 (13.2%)	14 (42.4%)	29 (13.9%)	12 (40.0%)	< 0.01
Prenatal antidepressant use^c^	18 (3.8%)	5 (15.2%)	5 (2.4%)	3 (10.0%)	< 0.01

^a^Shown as median (Q1, Q3) or N (column percent)

^b^Kruskal Wallis test for continuous variables, Chi Square for categorical

^c^From last menstrual period to delivery date

Of the 751 pregnancies included, 751 had maternal LTL and mtDNAcn results, 323 had neonatal LTL, and 337 had neonatal mtDNAcn. In multivariable analyses, mothers with high depressive symptoms alone had shorter maternal LTL (T/S ratio ß = −0.07, 95% confidence interval (CI) −0.13, −0.02] as did mothers classified with moderate-high anxiety symptoms alone (T/S ratio ß = −0.04, 95% CI −0.08, −0.01, Table 3) compared to mothers with low anxiety and low depressive symptoms.

**Table 3:** Adjusted associations of pregnancy-related anxiety and/or high prenatal depressive symptoms with maternal and neonatal biomarkers (*N* = 751 pregnancies)

	**Maternal biomarkers**		**Neonatal biomarkers**	
	**Leukocyte telomere length**^**a,b**^ ***N* = 751**	**Mitochondrial DNA copy number**^**a,b**^ ***N* = 751**	**Leukocyte telomere length**^**a,c**^ ***N* = 323**	**Mitochondrial DNA copy number**^**a,c**^ ***N* = 337**
Low PrAS^d^, EPDS^d^ < 13 (Referent, *N* = 479 maternal, *N* = 214 neonatal)^d^				
Mean (standard deviation)	0.70 (0.28)	1.02 (0.29)	1.30 (0.73)	1.01 (0.25)
EPDS ≥ 13 alone (*N* = 33 maternal, *N* = 13 neonatal)				
Mean (standard deviation)	0.63 (0.14)	1.01 (0.38)	1.09 (0.37)	1.18 (0.42)
ß effect size (95% CI)	−0.07 (−0.13, −0.02)	0.01 (−0.09, 0.11)	−0.20 (−0.45, 0.05)	0.11 (0.01, 0.21)
Moderate-high PrAS alone (*N* = 209 maternal, *N* = 98 neonatal)^e^				
Mean (standard deviation)	0.66 (0.18)	1.08 (0.35)	1.32 (0.98)	1.01 (0.24)
ß effect size (95% CI)	−0.04 (−0.08, −0.01)	0.01 (−0.02, 0.05)	−0.05 (−0.22, 0.11)	−0.00 (−0.04, 0.04)
Moderate to high PrAS and EPDS ≥ 13 (*N* = 30 maternal, *N* = 15 neonatal)				
Mean (standard deviation)	0.67 (0.19)	1.13 (0.34)	1.53 (1.64)	1.05 (0.17)
ß (95% CI)	−0.03 (−0.12, 0.05)	0.02 (−0.05, 0.10)	0.36 (−0.44, 1.16)	0.03 (−0.03, 0.09)

^a^Leukocyte telomere length units are T/S ratio, mitochondrial DNA copy number units are sample/population average

^b^Linear regression using generalized estimating equations accounting for repeat births, adjusted for maternal age at enrollment, body mass index pre-pregnancy, nulliparity, education, income level, smoking status, race and ethnicity as a social construct, use of antidepressant medication antepartum, and analytic plate

^c^Linear regression using generalized estimating equations accounting for repeat births, adjusted for maternal age at enrollment, body mass index pre-pregnancy, nulliparity, education, income level, smoking status, use of antidepressant medication antepartum, gestational age at delivery, sex, birthweight z-score for age and sex, and analytic plate. Numbers provided in column header are the total N included in the multivariable models

^d^Pregnancy-related anxiety (PrAS), Edinburgh Postnatal Depression Scale (EPDS). See text for details

^e^N = 479 maternal samples, 214 neonatal LTL, 212 neonatal mtDNAcn

^f^N = 209 maternal samples, 98 neonatal LTL, and 97 neonatal mtDNAcn

**Fig. 2:**
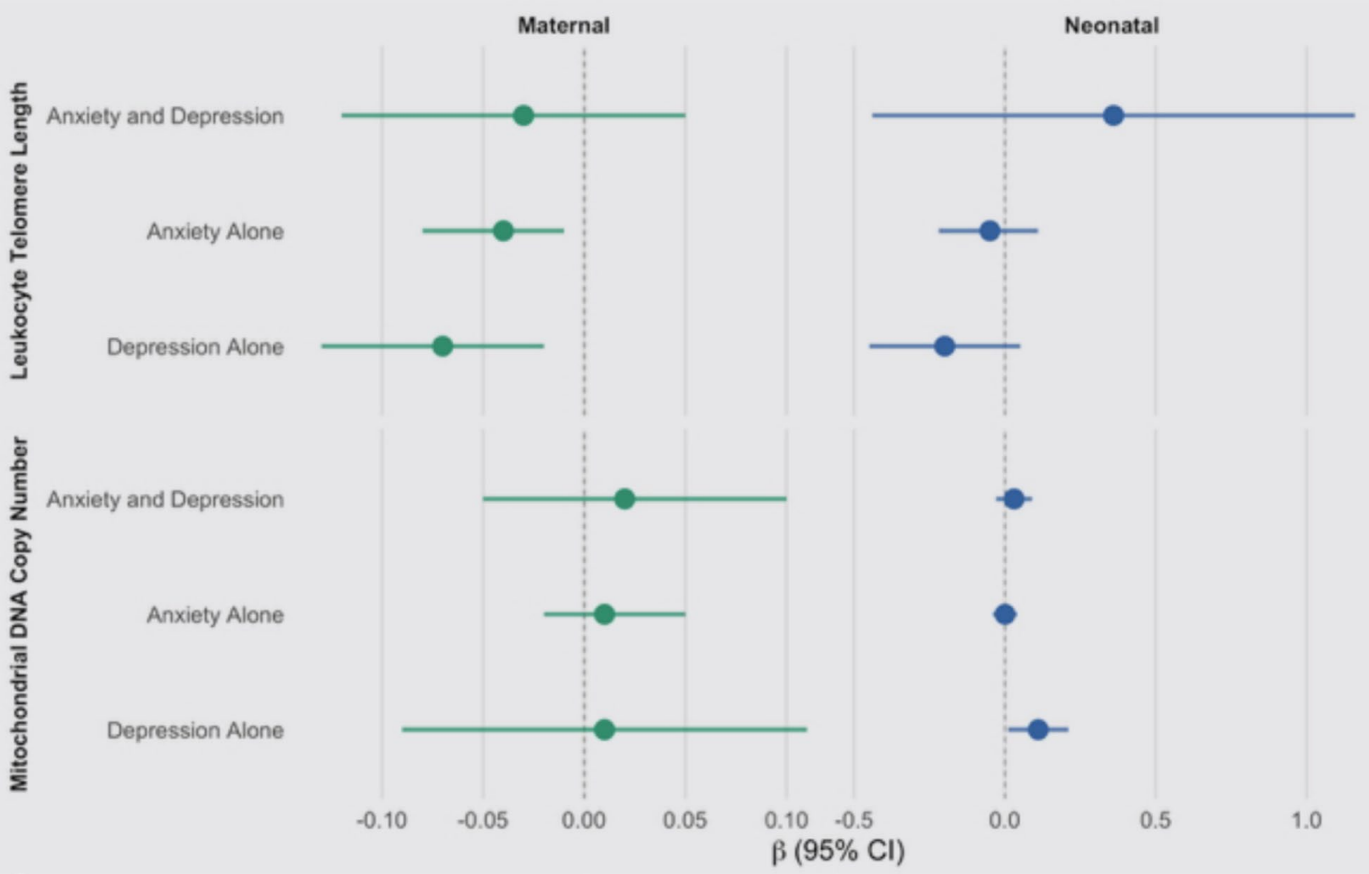
Forest plots of adjusted associations of pregnancy-related anxiety and/or high depressive symptoms with maternal and neonatal biomarkers of aging and oxidative stress. *Legend: For all analyses the reference category is low Pregnancy-related Anxiety Score and Edinburgh Postnatal Depression Scale (EPDS) score* < *13*

Among 337 mother-infant pairs with complete data for neonatal analyses, mothers classified as having high depressive symptoms alone had children with higher neonatal cord blood mtDNAcn (ß = 0.11, 95% CI 0.01, 0.21). The beta coefficient for the association between prenatal depressive symptoms and cord blood LTL in neonates was negative (similar to maternal LTL results), but the estimate was imprecise (95% CI −0.45, 0.05) and not statistically significant. Forest plots for adjusted associations from all analyses performed are shown in Fig. 2.

Next, we conducted four sensitivity analyses. We re-ran the models excluding participants on antidepressant medications at any time during pregnancy, restricting to 1 observation per participant (keeping their first pregnancy in the cohort), and restricting to pregnancies where 1) the mother completed the PrAS and EPDS in the first or second trimesters only (< 28 weeks). The LTL results from the primary analyses were mostly robust across analyses, but results for neonatal mtDNAcn were attenuated in the analysis restricted to EPDS and PrAS before 28 weeks (Supplementary Table 3). In the last sensitivity analysis evaluating continuous mental health questionnaire scores, the EPDS scores were log transformed. The interaction term for neonatal mtDNAcn was significant (*p* = 0.01) while the others were not (*p* = 0.78 for maternal LTL, 0.74 for maternal mtDNAcn, and 0.63 for neonatal LTL).

## Discussion

In this well-characterized pregnancy cohort, high prenatal depressive symptoms and moderate-high pregnancy-related anxiety symptoms were each independently associated with molecular signatures of accelerated maternal biological aging and neonatal oxidative stress. Both the depressive symptom-alone and pregnancy-related anxiety-alone groups had shorter maternal LTL, while neonates born to mothers with depressive symptoms alone had elevated cord blood mitochondrial DNA copy number. Shorter LTL was also consistently observed in neonatal cord blood with maternal depressive symptoms alone, but this association was statistically imprecise. We did not identify biological aging changes when depressive and pregnancy-related anxiety symptoms coexisted, though this was also the smallest subgroup. These findings suggest that there is a biological effect of maternal psychological symptoms that may have implications on long term maternal-child health, highlighting the need to address and improve mental health as part of routine obstetric care.

Our results advance prior literature on the biologic consequences of mental health conditions in mother-infant dyads. While limited, prior studies have suggested that maternal psychological stress is associated with increased inflammation and oxidative stress in maternal blood during pregnancy, upregulation of genes controlling inflammatory and beta-adrenergic pathways [27, 44], and accelerated aging markers, including mitochondrial DNA copy number, in the placenta [45, 46]. However, there has been heterogeneity in ascertainment of stress exposures and biologic outcomes in many prior studies [13, 47]; further, dyadic mother-infant biologic effects have rarely been included simultaneously, and pregnancy-related anxiety has historically been overlooked. Our findings extend this body of work by showing that depression and pregnancy-related anxiety symptoms are each independently associated with maternal and neonatal biomarkers of aging and oxidative stress responses.

Additionally, the majority of the prior literature has focused on fetal programming – linking maternal stress to neonatal biologic and later-life childhood outcomes – rather than on maternal biomarkers [11, 12, 17, 48, 49]. For example, in their literature review, Van den Bergh et. al [12]. identified several high quality studies relating maternal subjective stress or depression/anxiety to biomarkers of stress or aging in the neonate, including shortened LTL [12]. We identified an association between maternal depressive symptoms and cord blood mtDNAcn, which has been less well characterized in the context of maternal mental health. Both increases and decreases in neonatal mitochondrial DNA copy number can contribute to developmental origins of health and disease [17]. High or low mtDNAcn might be a biomarker of maladaptive stress responses [15, 50]; given our findings, further research should test long-term consequences of variation in this biomarker at birth.

The biological mechanisms underlying these results are likely multifactorial. Psychosocial stress activates the hypothalamic–pituitary–adrenal (HPA) axis and alters inflammatory cytokine profiles in ways that are exacerbated by gestational hormone shifts [51, 52]. These pathways may accelerate biologic aging via common underling pathways that manifest as telomere erosion and mitochondrial dysfunction [12, 39]. Our results align with this conceptual model. It was notable that the LTL shortening effects from depressive symptoms alone were not seen when restricting the analysis to pregnancies unexposed to antidepressant medications, while they persisted in the setting of pregnancy-related anxiety; this may suggest that medications could be a proxy for psychiatric disease severity, or that other biological changes may be occurring in the setting of antidepressant medication use [33]. The variation in mtDNAcn results when restricting to EPDS and PrAS before 28 weeks may suggest that depressive or anxiety symptoms at different stages may have differential effects on mtDNAcn. These questions should be addressed in future research.

Our findings reinforce American College of Obstetricians and Gynecologists (ACOG) guidelines recommending serial screening for both depression and anxiety in pregnancy, as we have demonstrated that these symptoms can each have direct biological manifestations in the perinatal period for both mother and child [2, 31]. Unfortunately, screening implementation remains suboptimal, with anxiety often overlooked. Notably, 28% of our sample screened positive for moderate-high pregnancy-related anxiety symptoms that were biologically active (manifested by shorter maternal LTL); these symptoms would have been missed using EPDS-based screening alone. Broader awareness and utilization of EPDS anxiety subscales or complimentary questionnaires may improve detection [53–55]. It was also notable that our prevalence of high depressive symptoms alone (4%) was lower than what has been reported in contemporary literature; this may be due to a stringent, high EPDS cutoff, or to historical differences in prevalence rates.

Awareness of the biologic consequences of untreated perinatal mental health symptoms takes on added urgency in light of recent questions surrounding the safety of antidepressant medication use in pregnancy [28]; in our sensitivity analysis, we identified a persistent link between mental health symptoms and maternal and neonatal stress biomarkers even after excluding participants on antidepressant medications, underscoring that symptoms are a primary driver of the biologic ramifications we assessed. Early identification and management of maternal mental health symptoms may represent a modifiable pathway to reduce the biological burden of psychosocial stress during pregnancy [25, 56].

Future work should explore the long-term implications of accelerated maternal and neonatal biomarkers of stress and aging, including their prenatal and postpartum trajectories and relationship to both pregnancy and later health outcomes. For instance, accelerated biological age during pregnancy has been associated with adverse outcomes including preterm birth and low birthweight, and may predict later-life cardiometabolic disease [57–61]. Biomarkers such as LTL and mtDNAcn provide an important window into cellular stress and repair capacity, but further research is needed to understand whether these changes persist, resolve, or amplify over time in this population. Expansion to more diverse and contemporary cohorts would also improve generalizability of our results. Interventional studies – including psychotherapy, resilience-building, and medication-based treatments – should evaluate whether these approaches can prevent or reverse biological aging signals in this population. Evidence suggesting that maternal resilience buffers against neonatal telomere attrition supports these endeavors [62]. As precision medicine evolves, incorporating biomarkers into risk stratification models may allow for more personalized antenatal care.

Our strengths include the use of a prospective cohort with rich phenotyping, validated instruments for mental health, assessment of anxiety and depression symptoms as distinct entities, and simultaneous analysis of mother-infant dyads in a relatively large cohort. Importantly, our results were robust even when excluding those on antidepressant medications – particularly for pregnancy-related anxiety symptoms [33]. Biomarkers were processed with rigorous laboratory protocols and confounders were carefully selected to minimize bias. However, limitations include smaller sample sizes for subgroup analyses – particularly those with combined pregnancy-related anxiety and depression – as well as the potential for Type 1 error. The cohort’s demographic composition may limit generalizability but does provide internal validity for biomarkers. The biomarker values for this study are interpreted as relative to this study cohort due to the process by which they were originally analyzed [30, 33], and may not be directly comparable to other study populations. While this may also limit generalizability, it does enhance internal validity for our study. The differences between our included cohort and the larger number of Project Viva participants who were excluded from our analysis may also limit generalizability of our findings. Our measure of anxiety was specific to pregnancy-related anxiety, which may not capture the full spectrum of clinical anxiety that may have been present. The observational nature of our study, as well as the timing of the mental health assessments relative to the collection of the maternal biological samples, limits causal inferences, but points to opportunities for future, prospective research.

High depressive and moderate-high pregnancy-related anxiety symptoms during pregnancy are associated with accelerated maternal biologic aging and increased neonatal oxidative stress biomarkers. These findings strengthen the biologic rationale for broader and nuanced mental health screening and treatment during pregnancy. Future research should elucidate mechanisms, long-term trajectories, and effective treatment strategies that could address these associations, towards the goal of improving maternal and child health.

## Supplementary Information

Supplementary Material 1.

## Data Availability

Deidentified datasets used and/or analyzed during the current study may be requested from Project Viva.

## Abbreviations

LTL: Leukocyte telomere length
MtDNAcn: Mitochondrial DNA copy number
EPDS: Edinburgh Postnatal Depression Scale
PrAS: Pregnancy-related anxiety scale
