# Supplementary material for: Association of depressive and/or pregnancy-related anxiety symptoms in pregnancy with maternal and neonatal biologic aging
Source: BMC Pregnancy Childbirth. 2026 Feb 17;26:321. doi: 10.1186/s12884-026-08810-1 (PMC13014748; doi:10.1186/s12884-026-08810-1)
Supplement: Supplementary file 1 — Supplementary Material 1. [file 12884_2026_8810_MOESM1_ESM.docx]

**Supplementary Table 1. Demographic and clinical characteristics among study participants versus those excluded from Project Viva cohort, 1999-2002 (N=2128).**

|  | **Excluded^a^**  **N = 1377** | **Included**  **N = 751** | **p-value^b^** |
| --- | --- | --- | --- |
| Median maternal age (years) | 32.0 (28.3, 35.1) | 32.5 (29.8, 35.7) | <0.01 |
| Median pre-pregnancy body mass index (BMI, kg/m^2^)^c^ | 23.5 (20.9, 27.5) | 23.5 (21.3, 26.7) | 0.78 |
| Self-reported race and ethnicity |  |  | <0.01 |
| Asian | 90 (6.7%) | 30 (4.0%) |  |
| Black | 281 (20.8%) | 67 (8.9%) |  |
| Hispanic | 109 (8.1%) | 45 (6.0%) |  |
| White | 819 (60.5%) | 580 (77.2%) |  |
| More than 1 race/ethnicity | 48 (3.6%) | 29 (3.9%) |  |
| Annual income >$70,000 | 661 (58.9%) | 485 (64.6) | 0.01 |
| College graduate | 795 (58.8%) | 565 (75.2%) | <0.01 |
| Nulliparous | 627 (45.5%) | 390 (51.9%) | 0.01 |
| Smoked tobacco early in pregnancy | 179 (13.2%) | 87 (11.6%) | 0.28 |
| Median gestational age at maternal blood draw | 9.9 (8.7, 11.1) | 9.6 (8.7, 10.7) | 0.10 |
| Median gestational age at delivery (weeks) | 39.7 (38.7, 40.6) | 39.9 (38.9, 40.7) | 0.01 |
| Median birthweight for age and sex (z-score) | 0.1 (-0.6, 0.8) | 0.2 (-0.4, 0.9) | <0.01 |
| Female infant | 658 (47.8%) | 374 (49.8%) | 0.39 |

*^a^ Shown as median (Q1, Q3) or N (column percent).*

*^b^ Wilcoxon rank sum test for continuous variables, Chi Square for categorical.*

*^c^ Missing values among all N=2128 as follows: N=16 BMI, N=24 college graduate, N=254 annual income, N=21 smoking status, N=24 race and ethnicity, N=1191 gestational age at blood draw (missing due to LTL and/or mtDNAcn results), and N=1 for infant infant birthweight for age/sex z-score.*

**Supplementary Table 2. Demographic and clinical characteristics among study participants with neonatal cord blood samples available, by mental health status (N=340).**

|  | **Low PrAS, EPDS <13^a^**  **N = 214** | **EPDS >13**  **alone**  **N = 13** | **Moderate-high PrAS alone**  **N = 98** | **Both moderate-high PrAS and EPDS>13**  **N = 15** |
| --- | --- | --- | --- | --- |
| Median maternal age (years) | 32.6  (29.9, 35.6) | 33.2  (29.1, 35.7) | 32.0  (29.3, 35.9) | 30.4  (27.1, 33.5) |
| Median pre-pregnancy body mass index (kg/m^2^) | 23.6  (21.6, 26.9) | 21.8  (20.4, 23.5) | 23.1  (20.6, 27.5) | 23.3  (21.4, 26.7) |
| Self-reported race and ethnicity |  |  |  |  |
| Asian | 6 (2.8%) | 3 (23.1%) | 3 (3.1%) | 0 |
| Black | 13 (6.1%) | 1 (7.7%) | 10 (10.2%) | 5 (33.3%) |
| Hispanic | 5 (2.4%) | 0 | 13 (13.3%) | 3 (20.0%) |
| White | 181 (84.6%) | 8 (61.5%) | 68 (69.4%) | 7 (46.7%) |
| More than 1 | 9 (4.2%) | 1 (7.7%) | 4 (4.1%) | 0 |
| Annual income >$70,000 | 147 (68.7%) | 6 (46.2%) | 61 (62.2%) | 5 (33.3%) |
| College graduate | 165 (77.1%) | 8 (61.5%) | 65 (66.3%) | 10 (66.7%) |
| Nulliparous | 99 (46.3%) | 8 (61.5%) | 62 (63.3%) | 8 (53.3%) |
| Smoked tobacco early in pregnancy | 20 (9.4%) | 2 (15.4%) | 14 (14.3%) | 1 (6.7%) |
| Median gestational age at maternal blood draw | 9.6 (8.7, 10.7) | 9.7 (9.3, 10.9) | 9.7 (8.8, 10.6) | 9.3 (8.6, 11.3) |
| Median gestational age at delivery (weeks) | 40.0  (39.0, 40.9) | 39.7  (38.7, 40.4) | 39.9  (38.9, 40.7) | 39.9  (39.4, 41.0) |
| Median birthweight for age and sex (z-score) | 0.4 (-0.2, 1.1) | 0.3 (-0.2, 0.7) | 0.1 (-0.5, 0.7) | -0.3 (-0.6, 0.7) |
| Female infant | 107 (50.0%) | 4 (30.8%) | 51 (52.0%) | 8 (53.3%) |
| Median PrAS score |  |  |  |  |
| Low | 214 (100.0%) | 13 (100.0%) | 0 | 0 |
| Moderate | 0 | 0 | 76 (77.6%) | 10 (66.7%) |
| High | 0 | 0 | 22 (22.5%) | 5 (33.3%) |
| Median EPDS score | 3.5 (1.0, 6.0) | 14 (13, 15) | 4 (2, 7) | 17 (15, 20) |
| Median gestational age at mental health assessment |  |  |  |  |
| PrAS | 9.6 (8.7, 10.7) | 9.7 (9.3, 10.9) | 9.7 (8.8, 10.6) | 9.1 (8.6, 11.3) |
| EPDS | 27.7  (26.6, 28.6) | 27.3  (27.1, 28.3) | 27.8  (26.7, 28.7) | 27.3  (26.4, 28.9) |

*^a^ Shown as median (Q1, Q3) or N (column percent). Edinburgh Postnatal Depression Scale (EPDS). Pregnancy-related Anxiety Score (PrAS). See text for details.*

**Supplementary Table 3.** Sensitivity analyses demonstrating adjusted associations of maternal pregnancy-related anxiety (PrAS) and depressive symptoms [Edinburgh Postnatal Depression Scale (EPDS)] during pregnancy with maternal and neonatal biomarkers.

|  | **Maternal biomarkers** | | **Neonatal biomarkers** | |
| --- | --- | --- | --- | --- |
|  | **Leukocyte telomere length**  ß (95% CI)^a^ | **Mitochondrial DNA copy number**  ß (95% CI)^a^ | **Leukocyte telomere length**  ß (95% CI)^b^ | **Mitochondrial DNA copy number**  ß (95% CI)^b^ |
| **Excluding participants who used antidepressant medications (N=720 pregnancies)** | | | | |
| Low PrAS, EPDS <13 (N=461) | Referent | | | |
| EPDS > 13 alone (N=28) | -0.05  (-0.10, 0.01) | 0.02  (-0.09, 0.13) | -0.20  (-0.46, 0.07) | 0.11^c^  (0.00, 0.22) |
| Moderate to high PrAS alone (N=204) | -0.04^c^  (-0.08, -0.01) | 0.02  (-0.02, 0.05) | -0.04  (-0.21, 0.12) | -0.01  (-0.05, 0.03) |
| Moderate to high PrAS and EPDS >13 (N=27) | -0.01  (-0.10, 0.07) | 0.00  (-0.08, 0.08) | 0.42  (-0.50, 1.34) | 0.03  (-0.04, 0.09) |
| **Restricting to first birth in cohort (N=744 pregnancies)** | | | | |
| Low PrAS, EPDS <13  (N=473) | Referent | | | |
| EPDS > 13 alone (N=32) | -0.07 ^c^  (-0.12,-0.01) | 0.01  (-0.09, 0.11) | -0.19  (-0.44, 0.06) | 0.11^c^  (0.01, 0.21) |
| Moderate to high PrAS alone (N=209) | -0.04 ^c^  (-0.08,-0.01) | 0.01  (-0.02, 0.05) | -0.04  (-0.21, 0.13) | 0.00  (-0.04, 0.04) |
| Moderate to high PrAS and EPDS >13 (N=30) | -0.04 (-0.12, 0.05) | 0.02  (-0.06, 0.09) | 0.38  (-0.42, 1.18) | 0.03  (-0.03, 0.09) |
| **Restricting to EPDS and PRAS done <28 weeks gestation (N=424 pregnancies)** | | | | |
| Low PrAS, EPDS <13  (N=276) | Referent | | | |
| EPDS > 13 alone (N=20) | -0.09^c^  (-0.16, -0.01) | 0.02  (-0.13, 0.16) | -0.16  (-0.54, 0.21) | 0.04  (-0.04, 0.12) |
| Moderate to high PrAS alone (N=108) | -0.07^c^  (-0.12, -0.02) | -0.01  (-0.06, 0.04) | 0.08  (-0.16, 0.31) | -0.02  (-0.07, 0.04) |
| Moderate to high PrAS and EPDS >13 (N=20) | -0.06  (-0.16, 0.04) | 0.10^c^  (0.02, 0.18) | 0.69  (-0.57, 1.94) | 0.02  (-0.07, 0.11) |

*^a^ Linear regression using generalized estimating equations accounting for repeat births, adjusted for maternal age at enrollment, body mass index pre-pregnancy, nulliparity, education, income level, smoking status, race and ethnicity as a social construct, and analytic plate.*

*^b^ Linear regression using generalized estimating equations accounting for repeat births, adjusted for maternal age at enrollment, body mass index pre-pregnancy, nulliparity, education, income level, smoking status, gestational age at delivery, sex, birthweight z-score for age and sex, and analytic plate. Numbers provided in column header are the total N included in the multivariable models for the maternal biomarkers.*

*^c^ Statistically significant at p<0.05*
